# Human Sperm Centrosome: From Current Evidence to Future Perspectives—A Systematic Review

**DOI:** 10.3390/life16060921

**Published:** 2026-05-30

**Authors:** Alessandra Parrella, Llanos Medrano, Jon Aizpurua, María José Gómez-Torres

**Affiliations:** 1 IVF Life, Reproductive Medicine, 03540 Alicante, Spain; a.parrella@ivf-spain.com (A.P.); ll.medrano@ivf-spain.com (L.M.); 2 Cátedra Human Fertility, Facultad de Ciencias, Universidad de Alicante, 03690 Alicante, Spain; j.aizpurua@ivfhealthholding.com; 3 Departamento de Biotecnología, Facultad de Ciencias, Universidad de Alicante, 03690 Alicante, Spain

**Keywords:** centrosome, human, infertility, human spermatozoa

## Abstract

Background: The sperm centrosome is a key organelle composed of two centrioles surrounded by pericentriolar material and is essential for sperm aster formation and first mitotic spindle formation after sperm entry into the oocyte, thereby supporting early embryonic development. Methods: This systematic review evaluated 83 publications published between 1986 and 2026 to provide an updated overview of the human sperm centrosome, including its structural organization, protein composition, role in reproductive success, and association with embryonic abnormalities. Results: The available evidence indicates that centrosome-associated proteins such as centrin, γ-tubulin, and pericentrin are critical for spindle assembly, microtubule nucleation, and centriole duplication, and that alterations in their expression or localization are associated with poor sperm quality and reduced fertilization potential. Centrosomal dysfunction has also been linked to fertilization failure despite intracytoplasmic sperm injection, abnormal embryo cleavage, and possible embryonic aneuploidy. Although diagnostic approaches have improved, targeted treatments remain limited. Conclusions: These findings highlight the clinical relevance of sperm centrosomal integrity and support the need for further research into its diagnostic and therapeutic implications.

## 1. Introduction

The sperm centrosome is a specialized organelle that plays a pivotal role in the early stages of embryo development. As the primary microtubule-organizing center (MTOC), it is involved in critical processes such as sperm aster formation, pronuclear migration, and assembly of the mitotic spindle, all of which are essential for the first cell division. In humans, this centrosomal structure is exclusively inherited from the sperm, representing a unique paternal contribution to the developing embryo [1,2,3]. Although the oocyte provides the cytoplasm necessary for centrosome reconstitution, it lacks centrioles due to their loss during oogenesis [1,4]. Structurally, the centrosome consists of a canonical proximal centriole and an atypical distal centriole, both embedded within segmented columns that represent modified pericentriolar material [5,6]. Among these, the most studied are γ-tubulin, which initiates microtubule nucleation, pericentrin, which anchors associated proteins during mitosis, and centrin, a calcium-binding protein involved in centriole duplication and contractile function [7]. Studies indicate that centrosomal dysfunction can impair key events that occur following fertilization. These disruptions may result in failed aster formation, irregular cell division, blastomere fragmentation, and poor embryo compaction, thereby decreasing the chances of successful implantation and pregnancy [7,8]. This systematic review provides a comprehensive analysis of the human sperm centrosome, including its structure and function, its role in fertilization, embryo development, aneuploidy, and the genes involved in its regulation. Additionally, we investigate emerging diagnostic strategies and therapeutic approaches designed to address centrosomal dysfunction and enhance reproductive outcomes.

## 2. Materials and Methods

### 2.1. Search Strategy

This systematic review was conducted in accordance with the PRISMA 2020 (Preferred Reporting Items for Systematic Reviews and Meta-Analyses) guidelines [9]. To ensure accuracy in bibliometric and bibliographic analysis, we conducted an extensive search of online databases relevant to the research topic. The search strategy included combinations of the following terms: (“centrosome” OR “centrosomal”) AND (“human sperm” OR “spermatozoa”) AND (“infertility” OR “fertilization”), applied to title, abstract, and keyword fields. Although additional related terms such as ‘centriole,’ ‘MTOC,’ and ‘sperm aster’ were not explicitly included in the search string, relevant studies using these terms were captured through the use of broader keywords and manual reference screening. The search included studies published between January 1986 and January 2026. The selected databases for this purpose were PubMed (https://pubmed.ncbi.nlm.nih.gov, accessed on 11 April 2026), Web of Science (WOS) (https://www.webofscience.com/wos/woscc/basic-search, accessed on 11 April 2026), and Scopus (https://www.scopus.com/, accessed on 11 April 2026). In the Web of Science database, we utilized the “Search all databases” option and applied the field tag “Topic” to refine the search. On the other hand, for the Scopus database, we employed the “Article title, Abstract, Keywords” field tag during the search. These choices were made to optimize the search process and ensure that relevant articles were included in the analysis. By leveraging these prominent scientific databases and employing specific search parameters, we aimed to gather a comprehensive and representative set of articles for our study, which supports the strength and credibility of the findings. This review was not registered, and no protocol was prepared.

### 2.2. Study Selection and Analysis of Data

Microsoft Excel was used to collect and compile results from the literature search, and duplicate articles were identified using both electronic and manual methods. All studies reporting on the centrosome in human spermatozoa were considered during the screening process. Additionally, to construct the final database, the following exclusion criteria were applied: (i) studies not solely focused on the centrosome in humans, (ii) studies not related to reproductive biology, (iii) studies outside the scope of the review, (iv) studies not published in English, (v) studies that were inaccessible or (vi) lacked full-text availability, (vii) studies where the centrosome was not the main topic. The inclusion and exclusion criteria were defined by MJG-T and AP. Titles and abstracts of all retrieved records were independently screened by two reviewers (MJG-T and AP). Full-text articles were obtained for studies considered potentially eligible, and were subsequently assessed according to predefined inclusion and exclusion criteria. Any discrepancies were resolved through discussion and consensus between the two reviewers. In addition, the reference lists of included studies were manually screened to identify any further relevant articles not captured by the electronic search. The systematic review ultimately included 83 articles.

### 2.3. Data Extraction

The data extraction process consisted of collecting the main information from each included study. Specifically, the following information was extracted: title, authors, year of publication, source title, affiliation, country, language, study type (e.g., case report, cohort study, review), and main findings. To ensure consistency and accuracy, a standardized data extraction sheet was used. Data extraction was performed by AP and MJG-T, and any discrepancies were resolved through discussion and consensus. All data reported in Supplementary Table S1 were verified against the original publications before submission. A formal risk of bias assessment was not performed due to the heterogeneity of the included studies in terms of study design, experimental approaches, and reported outcomes. Similarly, no formal assessment of reporting bias or certainty of evidence was undertaken.

## 3. Results

### 3.1. Compilation of Relevant Bibliographic Sources

**Figure 1:**
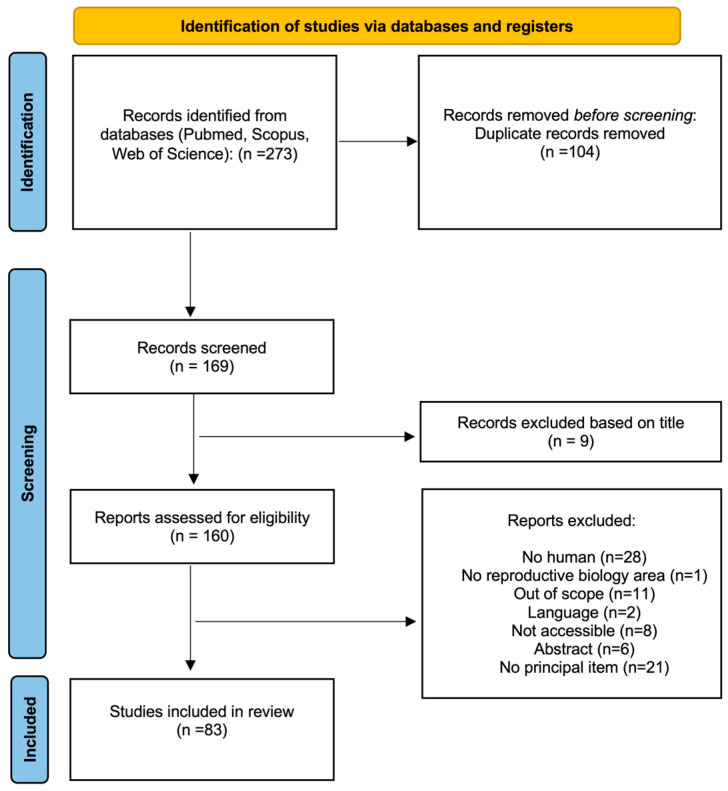
PRISMA 2020 flow diagram of the study selection process [9].

The literature search was performed using predefined keywords across three databases: PubMed, Scopus, and Web of Science. This initial search yielded 100 records from PubMed, 107 from Scopus, and 53 from Web of Science. Additionally, 13 articles were identified through manual searching, resulting in a total of 273 records. After the removal of duplicates, 169 unique records remained for screening. During the screening phase, 9 records were excluded based on the title as they were not relevant to the centrosome or its role in human spermatozoa. The remaining 160 reports were assessed for eligibility. Following the application of predefined inclusion and exclusion criteria, 77 articles were excluded. Ultimately, 83 studies were included in this review, representing 30.4% of the initially identified records. Of these, 40 were cohort studies (48.2%), 15 were case reports (18.1%), and 28 were reviews (33.7%) (Figure 1). A detailed summary of all included studies is provided in Supplementary Table S1.

### 3.2. Bibliometric Analysis

**Figure 2:**
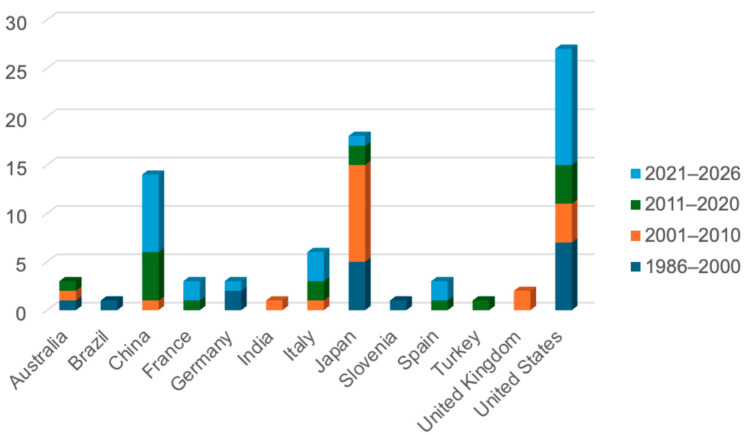
Number of publications on the human sperm centrosome by country, grouped by time periods (1986–2000, 2001–2010, 2011–2020, and 2021–2026).

**Figure 3:**
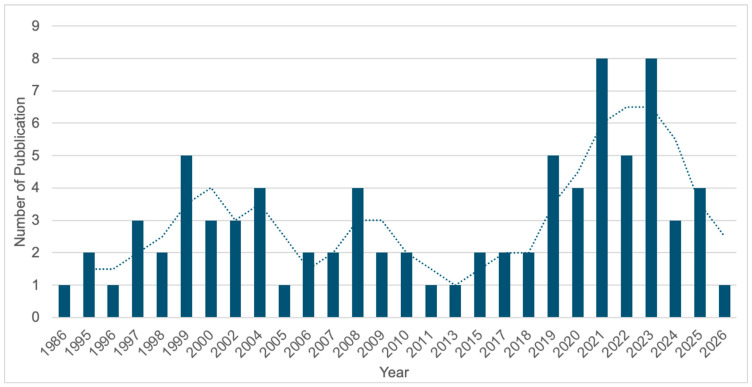
Annual number of scientific publications related to the centrosome in human spermatozoa. The dashed line represents the publication trend over time.

The article search was carried out from 1986 to 2026. The analysis of 83 articles revealed that the United States led with 27 publications (32.5%), underscoring its prominent role in advancing this field. Japan followed with 18 publications (21.7%), while China contributed 14 studies (16.9%), reflecting a strong and growing investment in centrosome research. Italy accounted for 6 publications (7.2%). Moderate contributions came from Australia, France, Germany, and Spain, each with 3 studies (3.6%), while the United Kingdom contributed 2 studies (2.4%). India, Brazil, Turkey, and Slovenia each had 1 publication (1.2%) (Figure 2). The years 2021 and 2023 recorded the highest number of publications, with 8 articles each (9.6%), followed by 1999, 2019, and 2022, each with 5 articles (6.0%). Years including 2004, 2008, 2020, and 2025 contributed 4 publications each (4.8%), while 1997, 2000, 2002, and 2024 showed moderate activity with 3 publications each (3.6%). Several years, including 1995, 1998, 2006, 2007, 2009, 2010, 2015, 2017, and 2018, each recorded 2 publications (2.4%). Conversely, 1986, 1996, 2005, 2011, 2013, and 2026 showed the lowest publication output, with only 1 article each (1.2%). Notably, the data for 2026 represent a partial year (up to April), which may underestimate the total number of publications (Figure 3). The upward trend in publication output, particularly in the last decade, suggests increasing global interest, likely driven by advances in molecular biology, imaging techniques, and assisted reproductive technologies. However, the variability in annual publication volume could be attributed to the fact that centrosome assessment remains primarily diagnostic, as there are currently no standardized or widely accepted therapeutic interventions available to address centrosomal dysfunction in the context of male infertility.

### 3.3. Bibliographical Analysis

The articles included explore the role of the centrosome in human reproduction, with a particular focus on its structure, function, and clinical implications. These studies have investigated the localization and function of centrosomal proteins in human spermatozoa, examining how abnormalities affect sperm motility, fertilization, and early embryonic development [10,11,12]. Specific attention has been given to the involvement of the centrosome in oocyte activation, zygote formation, and first mitotic spindle assembly, processes essential for successful fertilization and embryo cleavage [13,14,15]. A subset of studies has analyzed centrosomal dysfunction in rare conditions such as globozoospermia, dysplasia of fibrous sheath, and Kartagener’s syndrome, which are often associated with poor fertilization outcomes [16,17,18]. Despite its critical role, centrosome analysis remains primarily diagnostic, as no standardized therapeutic interventions currently exist to treat sperm centrosomal defects. However, recent research has begun to explore molecular and gene expression profiles related to centrosome integrity and the potential impact on embryo aneuploidy and developmental arrest [19,20]. These findings highlight the growing interest in the centrosome as both a diagnostic marker and a potential target for future therapeutic strategies in male infertility.

### 3.4. Structural and Functional Characteristics of the Centrosome

The sperm centrosome is composed of four main structural elements: the proximal centriole (PC), the distal centriole (DC), and two accessory components, the capitulum and the striated columns, which together form the connecting piece. The capitulum, also referred to as the capitellum, is a poorly defined structure, described as a transient element that may disappear in mature spermatozoa and possibly derived from the fusion of striated columns. The striated columns consist of nine longitudinal, electron-dense fibers with a characteristic banded appearance and are interconnected by fine bridges. Both the capitulum and striated columns are thought to originate from centriolar structures and surrounding pericentriolar material (PCM) during spermiogenesis. Accordingly, the capitulum and striated columns are regarded as modified forms of pericentriolar material. They contain multiple centrosomal proteins and together form a scaffold-like structure that creates two distinct compartments accommodating the proximal and distal centrioles. The proximal centriole is a conserved, canonical structure composed of nine microtubule triplets and is considered analogous to the mother centriole. It also contains a transient structure known as the centriolar adjunct (CA), a remnant cilium that has been associated with sperm maturation and fertility outcomes [21]. Incomplete disassembly of the CA has been implicated in idiopathic male infertility (IMS) and early developmental arrest of the zygote. Garanina et al. investigated this phenomenon using transmission electron microscopy (TEM) to compare the combined length of the proximal centriole and centriolar adjunct (PC+CA) in sperm samples from two patients with IMS and five fertile individuals. Their analysis revealed that, while the CA in fertile donors was either fully or partially disassembled, consistent with normal sperm maturation, it remained notably elongated and intact in IMS cases. Therefore, the presence of CA structures may signify sperm immaturity and perhaps compromise fertilization efficacy [1,22]. In contrast, the distal centriole, also known as the flagellar basal body, is highly remodeled during spermiogenesis, adopting a fan-shaped configuration composed of nine microtubule doublets, with centriolar luminal proteins reorganized into rod-like structures. It connects directly to the axoneme and displays structural asymmetry, contributing to the dynamic properties of the sperm flagellum. Although previously thought to degenerate in mature spermatozoa, it is now recognized as a modified yet functionally relevant centriole [12,23]. In mature spermatozoa, the centrioles are embedded within a remodeled PCM, which serves as a scaffold for essential protein complexes involved in centrosomal organization [24]. Successful human fertilization depends on precise microtubule dynamics. The zygote inherits centrioles exclusively from the spermatozoon, comprising a canonical proximal centriole and an atypical distal centriole, which, together with pericentriolar material recruited from the oocyte, form the first functional centrosome. Initially, the two sperm-derived centrioles remain closely associated near the decondensing male pronucleus, contributing to a single centrosomal unit that facilitates the migration and apposition of the female and male pronuclei [1,2,25,26]. During the pronuclear stage, centrosome duplication is initiated, and by the time syngamy is completed, the centrosomal units are positioned at opposite poles of the zygote, where they orchestrate chromosome segregation during the initial mitotic event. At metaphase, chromosomes align at the spindle equator through interactions with spindle microtubules. Two spatially separate bipolar spindles have been observed in human zygotes during anaphase, each associated with a distinct group of chromosomes. During this stage, spindle microtubules mediate the separation of sister chromatids and move them to opposite sides of the dividing cell. Mitosis ends with the formation of two genetically identical daughter cells, each containing a centrosome composed of one pre-existing (mother) and one newly formed (daughter) centriole. These sequential steps, from centriole replication to centrosome maturation, reflect the transformation of the sperm-derived centriole into a fully functional centrosome within the early embryo [13,14,27,28,29,30,31].

### 3.5. Key Protein Components of the Centrosome

The centrosome relies on a complex protein network that supports its structural integrity and key functions such as microtubule nucleation and chromosome segregation [31]. In sperm, the sperm basal body contains more than 250 centrosome-related proteins involved in centriole structure, duplication, microtubule nucleation, and PCM organization [6]. Among these proteins, centrin (CETN) is particularly important for the maturation of centrioles. CETN proteins belong to a well-conserved group of EF-hand calcium-binding proteins that are found in the lumen of mature centrioles [7,32]. They contribute to a variety of cellular processes, including centrosome replication and the proper functioning of cilia and flagella. In healthy sperm, CETN is visualized as two distinct fluorescent signals at the base of the flagellum, marking the positions of both centrioles. However, in sperm from certain infertile individuals, this localization pattern is disrupted, with diffuse or misplaced centrin signals obscuring centriole identification [33]. Research by Hinduja et al. revealed that men with oligoasthenozoospermia (OA) displayed markedly reduced CETN levels compared to normozoospermic individuals. This reduction was confirmed using both Western blotting and ELISA, with the latter showing a statistically significant decline in centrin expression in the OA group (*p* < 0.05) [7,10]. Humans express four CETN paralogs, with CETN1 and CETN2 displaying approximately 84% sequence similarity. CETN2 is expressed earlier during spermatogenesis, whereas CETN1 is predominantly expressed in mature sperm, where it plays a key role in maintaining centrosome integrity and supporting the stability and formation of the distal centriole and flagellum [34]. Another key protein is γ-tubulin, which plays a fundamental role in microtubule nucleation at the centrosome. It does not polymerise into a unit like α- or β-tubulin, but as a component of the γ-tubulin ring complex (γ-TuRC), which serves as a template for microtubule polymerization at the centrosome. During fertilization, γ-tubulin provided by the sperm centrosome initiates microtubule assembly, leading to the formation of the sperm aster. As the structure develops, it recruits more maternal γ-tubulin, resulting in a robust microtubule network that facilitates the movement and alignment of the male and female pronuclei [7]. In OA men, significantly lower expression levels of α- and γ-tubulin have been observed when compared to normozoospermic controls. The reduction in tubulin proteins was statistically significant (*p* < 0.005), suggesting a compromised centrosomal function [10]. Schatten and Sun further emphasized that defects in γ-tubulin expression or its recruitment to the centrosome can hinder proper microtubule organization and fertilization capacity [11]. Researchers also investigated the formation of the human zygotic centrosome by analyzing the respective contributions of maternal and paternal components, using mammalian zygotes and human sperm incubated with Xenopus laevis egg extracts. Western blotting confirmed the paternal origin of γ-tubulin, while maternal γ-tubulin was actively recruited to the sperm centrosome upon fertilization. Conversely, centrin, although present in the proximal sperm centrosome and sensitive to calcium, was not observed in the reconstituted zygotic centrosome post-fertilization or after extract exposure [35]. An additional PCM-associated protein is Aurora-C, highly expressed in human testicular tissue and localizes to structures such as centromeres and the midbody throughout spermatogenesis. Its correct activity is necessary for maintaining chromosome alignment and completing cytokinesis in male germ cells. Abnormal expression or mutations in the *Aurora-C* gene have been linked to reproductive disorders such as polyploidy in sperm [24]. TSKS (testis-specific serine kinase substrate) is a protein that localizes specifically to the centrioles of post-meiotic spermatids, reaching peak expression during flagellogenesis. Notably, TSKS interacts with TSSK2, a testis-specific serine/threonine kinase also present in the sperm neck and equatorial segment, supporting the hypothesis that this kinase/substrate pair plays a critical role in spermiogenesis [36]. Pericentrin, a large coiled-coil protein, serves as a primary structural component of the PCM, essential for anchoring and organizing key proteins, particularly during mitosis. It exists in two forms created through alternative splicing, with the larger form, pericentrin B, featuring a C-terminal region that binds to calmodulin. However, the specific role of pericentrin in fertilized eggs remains uncertain [7]. The study by Fishman et al. challenges the classical view of centrosome reduction during spermatogenesis by demonstrating that the human DC is not eliminated but remodeled into an atypical yet functional structure. Through super-resolution microscopy and immunofluorescence, the researchers characterized the distribution of centrosomal proteins within the PC, DC, centriole, and PCM. Several proteins, such as CETN1/2, POC5, and members of the POC1 family, are distributed across both proximal and distal regions of the centriole. Within this group, POC1A and POC1B are particularly important for maintaining the architectural integrity and elongation of the centriole. Interestingly, POC1B was found to be more abundant in the distal portion, indicating it may play a specialized role in that substructure of the centrosome [12]. In contrast, while POC1A is not directly involved in the process of centriole duplication, it is crucial for maintaining the overall structural stability of the centriole [34]. Other key proteins involved in centrosome duplication, such as CEP63 and CPAP, were also found in both centrioles, supporting their potential role in zygotic centrosome inheritance. While classical PCM markers like γ-tubulin and pericentrin were not detected, other PCM-related proteins, such as CEP164 and CDK5RAP2, were localized to structures like the capitulum and striated columns of the sperm neck, indicating a specialized PCM configuration. Importantly, functional assays using Xenopus egg extracts showed that the atypical DC can still recruit γ-tubulin and support sperm aster formation, affirming its active role in initiating early embryonic development [12]. Turner et al. investigated the localization of the centriolar proteins CEP135 and CP110 in human and bovine spermatozoa. Using centriolar markers such as POC1B and tubulin, they confirmed the presence of two distinct foci corresponding to the proximal and distal centrioles. In human spermatozoa, CEP135 localizes to the striated columns and capitulum in proximity to the centrioles, with a predominant signal at the base of the proximal centriole and a weaker distribution near the distal centriole, supporting its role in microtubule assembly and centriole formation. In contrast, CP110, a centriole tip protein known to inhibit ciliogenesis, is also detected in adjacent structures, including the capitulum and striated columns. The redistribution of centriolar proteins to these specialized compartments is likely due to centrosome reduction and centriole reorganization during spermiogenesis [37,38]. Pericentriolar material 1 (PCM1) also contributes to centrosome organization. It is a key scaffolding protein of centriolar satellites, dynamic cytoplasmic granules that mediate the trafficking and delivery of proteins required for centrosome function and ciliogenesis. PCM1 interacts with centrosomal proteins and regulates their transport via intramanchette transport, a process essential for centrosome remodeling and axoneme biogenesis during spermiogenesis [39]. Together, these findings highlight the importance of regulatory pathways, such as the Aurora A–HP1γ axis, in maintaining centrosomal function, ensuring proper microtubule organization [40].

### 3.6. Experimental Assessment of the Sperm Centrosome

A range of experimental approaches has been developed to evaluate the integrity and functionality of the sperm centrosome, including its ability to form asters [41]. Following early developments in the field, Colombero et al. explored the localization of centrosomes using immunofluorescent labeling with xenogenic reagents. They tested three antibodies: rabbit anti-mitotic spindle protein (anti-MSP), rabbit polyclonal centriole-specific antibodies, and mouse monoclonal anti-MPM-2 targeting a centrosomal phosphoprotein. The anti-MPM-2 demonstrated the strongest labeling performance, detecting centrosomal components in 63% of spermatozoa. This study highlighted the potential of immunolabeling for evaluating centrosomal integrity and advancing diagnostic methodologies [42]. In parallel, the use of heterologous ICSI, where human sperm is injected into non-human mammalian oocytes, has emerged as a valuable model for investigating sperm centrosomal function, due to ethical and logistical constraints associated with human oocytes. Among these models, rabbit and bovine oocytes have been identified as superior to traditional models like mice and hamsters. Nonetheless, important biological differences between species must be carefully considered when interpreting results [43]. To better understand these differences, Terada et al. compared heterologous ICSI outcomes using hamster, rabbit, and bovine oocytes injected with sperm from both fertile and infertile men. While astral microtubules were absent in hamster oocytes, rabbit oocytes exhibited lower egg activation rates (30%) and reduced sperm aster formation rates. In contrast, bovine oocytes showed higher egg activation rates (83.3%) and sperm aster formation rates (60%), closely resembling the dynamics observed in human fertilization [44]. The same group employed rabbit oocytes in a heterologous ICSI assay to analyze sperm from infertile men. The results showed lower sperm aster formation rates (25.4 ± 14.8%) in infertile men compared to fertile individuals (35.0 ± 1.5%) and showed a positive correlation with embryonic cleavage rates, highlighting their relevance in Assisted Reproductive Technology (ART) [45]. Further studies using bovine oocytes demonstrated that sperm from infertile men exhibited significantly lower sperm aster formation rates (47.0%) compared to those from fertile men (66.1%). This reduction was associated with decreased embryonic cleavage and pregnancy rates. Notably, despite normal semen parameters and unaffected pronuclear formation rates, centrosomal dysfunction negatively impacted embryonic development and pregnancy success [46]. A compelling clinical case was reported by Terada et al., who employed artificial oocyte activation (AOA) in a patient with recurrent ICSI failure attributed to sperm centrosomal dysfunction. After seven unsuccessful ICSI attempts, AOA using a calcium ionophore resulted in a clinical pregnancy and the subsequent birth of a healthy infant. While oocyte activation rates were comparable to those of controls (97% vs. 100%), the patient’s sperm showed impaired microtubule organization, with a sperm aster formation rate of 48.5% compared to 69.6% in fertile controls [47]. More recently, Turner et al. developed a novel diagnostic tool, Fluorescence-Based Ratiometric Analysis of Sperm Centrioles (FRAC), to assess sperm centriole quality. Using biomarkers such as tubulin and POC1B, they demonstrated that a substantial proportion of infertile men exhibit altered centriole profiles, particularly in cases of abnormal sperm morphology, with defects observed in up to 78% of teratospermic patients compared with 14% in men with normal morphology. Furthermore, centriole quality declined with age [48]. This approach was further refined in men with unexplained infertility using additional biomarkers, including γ-tubulin, POC1B, and acetylated tubulin. FRAC successfully distinguished fertile from infertile individuals, revealing a higher frequency of abnormal centriole patterns in the infertility group (*p* = 0.0008), with acetylated tubulin emerging as the most frequently altered marker [49]. Subsequent studies have investigated the functional consequences of sperm centriolar defects identified by FRAC. In a pilot study, abnormal centriole profiles in spermatozoa were significantly associated with impaired zygote centrosome function, as assessed by nucleolus precursor body (NPB) polarization patterns. Embryos derived from spermatozoa with abnormal FRAC parameters exhibited a higher incidence of defective NPB polarization, indicating compromised centrosome activity during early embryonic development [25]. Finally, Amargant et al. introduced Xenopus egg extracts as an ex vivo system to assess sperm-induced bipolar spindle assembly. Human spermatozoa were able to induce bipolar spindle formation with efficiency comparable to controls (65.3% vs. 60.7%, *p* = 0.53). While overall spindle formation rates were similar between normozoospermic and asthenozoospermic samples, the latter showed a higher proportion of abnormal spindle structures (*p* = 0.010). These findings suggest that abnormal spindle morphology may reflect sperm quality rather than centrosomal defects [50].

### 3.7. Paternal Centrosome Contribution to Human Fertilization

Centrosomal dysfunction, including incomplete or truncated sperm aster formation or improper microtubule nucleation, can prevent proper pronuclear alignment and union of parental genomes. These defects often lead to fertilization failure, developmental arrest, or compromised embryo quality [1,13,14,30,51,52,53,54]. One of the pivotal roles of the sperm aster in successful fertilization was demonstrated in a landmark 1995 experiment using human and rhesus oocytes. In this study, oocytes treated with nocodazole, a microtubule-inhibiting compound, failed to form sperm aster microtubules or achieve pronuclear apposition. However, sibling oocytes injected with donor sperm achieved successful fertilization, whereas no fertilization occurred with the husband’s sperm, suggesting defects after sperm entry [2]. Building on this, another study investigated sperm centrosomal dysfunction in fertilization failure, distinguishing between men with severe oligoteratozoospermia following vasectomy reversal and those with idiopathic forms. Oocytes injected with sperm from vasectomy-reversal patients failed to form pronuclei, whereas those from idiopathic cases achieved fertilization but arrested at the single-cell stage. In contrast, donor sperm resulted in normal fertilization and embryonic progression. Immunofluorescence analysis of the unfertilized oocytes revealed sparse and truncated microtubules from the sperm centrosome, indicating impaired centrosomal function [52]. Navara et al. conducted one of the earliest comprehensive studies on the role of the paternally derived centrosome in human fertilization. Using immunohistochemical imaging of inseminated human and rhesus oocytes, they identified multiple stages where fertilization may arrest, associated with inadequate sperm aster formation and impaired pronuclear apposition. In some infertile patients, the sperm centrosome failed to properly nucleate and organize microtubules, resulting in unsuccessful fertilization despite normal sperm entry. The authors also suggested that the rate, size, and organization of the sperm aster could serve as a valuable marker of sperm quality. Moreover, semen from men with reduced fertility often contained spermatozoa incapable of forming asters, reflecting underlying centrosomal dysfunction [29]. Kovačić and Vlaisavljević investigated the cytological mechanisms underlying fertilization failure after ICSI by analyzing unfertilized human oocytes. Most oocytes were arrested at metaphase II and showed abnormalities in spindle integrity and sperm chromatin remodeling, including premature chromatin condensation (G1-PCC). In some cases, spermatozoa were absent, or chromatin decondensation was delayed. These findings suggest that when sperm fails to properly activate the oocyte, male pronucleus formation is impaired, likely due to oocyte-induced PCC and disrupted centrosomal activity [55]. Rawe et al. investigated centrosomal dysfunction in a patient with severe asthenoteratozoospermia as a cause of ICSI failure. Structural abnormalities, including acephalic spermatozoa and defective head–tail junctions, were observed. Functional assays using bovine oocytes showed reduced pronuclear formation and sperm aster development compared to controls. Clinically, ICSI initially failed to achieve syngamy or cleavage, and although stricter sperm selection improved fertilization and cleavage rates, no ongoing pregnancies were achieved [8]. The “easily decapitated” sperm defect is a structural abnormality of the sperm neck in which the head readily detaches from the tail. Ultrastructural studies have shown defects in the head–neck junction, with detached tails often retaining one or two centrioles within disorganized cytoplasmic structures. Although ICSI has occasionally resulted in pregnancies using intact or reassembled sperm, these abnormalities are typically associated with centrosome dysfunction and impaired embryo development. The genetic basis of this defect remains unclear, raising concerns about its use in ART [56]. Holstein et al. described a rare malformation involving sperm decapitation caused by a defect during early spermatid differentiation. Ultrastructural analysis of a testicular biopsy from an infertile patient revealed premature separation of the proximal and distal centrioles due to the absence of striated columns, leading to independent development of the sperm head and tail. As a result, most spermatozoa were decapitated, predominantly consisting of isolated tails, some of which retained motility [57]. Further insights into the importance of sperm structural integrity for centrosomal function were provided by Palermo et al., who investigated the developmental potential of human embryos following ICSI with mechanically dissected spermatozoa. In this study, 77 human oocytes were injected with dissected sperm components, including isolated heads, physically separated heads and tails, and isolated tails. Pronuclei were observed in 61% of oocytes injected with isolated heads, 64% with head–tail pairs, and 22% with isolated tails. Despite successful fertilization, a large number of embryos exhibited chromosomal defects. Indeed, fluorescence in situ hybridization (FISH) analysis revealed that only a few embryos derived from isolated head injections preserved a standard diploid chromosome complement, whereas embryos produced from separated head–tail pairs or isolated tails consistently showed chromosomal mosaicism. This could arise from damage to the sperm’s centriole, alterations in the PCM, or abnormal activation of the distal centriole, potentially resulting in spindle abnormalities such as multipolar formations [1]. This concept is supported by earlier studies in which only the sperm head was used for fertilization. Although fertilization itself was not impaired, subsequent embryonic development was severely compromised, as the sperm nucleus alone is insufficient to sustain the first zygotic division in humans. Genetic analysis of the resulting blastomeres revealed extensive chromosomal abnormalities and chaotic mosaicism [54,58]. On the other hand, Emery et al. demonstrated that successful pregnancy can still be achieved with ICSI in cases of easily decapitated spermatozoa, as long as the sperm head and midpiece are carefully realigned during injection. In their study, a motile sperm cell underwent unintentional decapitation, after which the midpiece was carefully introduced into the oocyte and aligned near the sperm head using ICSI. This approach resulted in a fertilization rate of around 63%, with 36% of the resulting embryos deemed high quality. The transfer of two embryos led to a successful pregnancy and the birth of a healthy baby. TEM analysis identified significant ultrastructural abnormalities in the sperm, such as damage to the basal plate and centrosomal components. Despite these defects, the presence of partially functional centrosomes and the accurate positioning of the midpiece likely contributed to the favorable developmental outcome [59]. Centrosome integrity appears to play an important role in fertilization and embryo development, highlighting the need for detailed analyses of centrosomal function in infertile individuals.

### 3.8. Centrosome Function During Embryo Cleavage

Several studies have examined the contribution of the sperm centrosome to the regulation of cleavage events following fertilization. The study by Sathananthan et al. analyzed over 200 human embryos using TEM and fluorescent microscopy to examine the role of the sperm centrosome in embryonic cleavage. The centrosome duplicates at the pronuclear stage and directs spindle formation throughout early divisions. Bipolar spindles were consistently observed, supporting normal cleavage progression, whereas centrosomal defects have been associated with abnormal cleavage [15,60]. Moomjy et al. examined the impact of sperm integrity on embryo cleavage and early development. Oocytes injected with intact spermatozoa developed normally, forming pronuclei and undergoing cleavage. However, oocytes injected with dissected spermatozoa (sperm head or head and tail separately) also formed pronuclei but showed an increased incidence of abnormal chromosome segregation. Notably, oocytes injected with heads and tails separately and those injected with isolated sperm tails failed to form a proper bipolar spindle, which may contribute to disrupted mitotic division and chromosomal mosaicism [61]. To more precisely evaluate centrosomal function, researchers have employed heterologous ICSI models involving bovine and rabbit oocytes. Terada et al. assessed sperm aster formation after ICSI, in which sperm samples from infertile men and fertile donors were injected into rabbit oocytes, and found that sperm from fertile donors showed higher aster formation rates than sperm from infertile patients. Importantly, sperm aster formation positively correlated with embryo cleavage rates, with higher aster formation observed in patients with better cleavage outcomes [45]. Similarly, Yoshimoto-Kakoi et al. analyzed sperm from infertile patients and fertile donors using heterologous ICSI with bovine oocytes to evaluate embryo cleavage. The study found that reduced sperm aster formation was associated with lower cleavage rates. Patients with high cleavage rates (>75%) exhibited significantly greater aster formation (57.0%) compared with those with lower cleavage rates (32.2%, *p* < 0.05) [46]. Chatzimeletiou et al. investigated cleavage-stage embryos from a patient with oligoasthenoteratozoospermia (OAT) and observed delayed or arrested embryonic development, accompanied by polyploidy and post-zygotic chromosome missegregation. These abnormalities were associated with defective centrosomal distribution and cytokinesis, leading to abnormal spindle organization and uneven chromosome segregation. The findings suggest that delayed zygotic division may result in tetraploidy, potentially due to monopolar spindle formation or failed cytokinesis [7]. The study by Tarozzi et al. explored the impact of sperm centrosomal function on early embryonic development and cleavage patterns in ART cycles. The research revealed that sperm centrosomal dysfunction may contribute to irregular cleavage patterns, slower embryonic division, and increased fragmentation, often associated with developmental arrest before the blastocyst stage. Additionally, the preimplantation genetic testing for aneuploidy (PGT-A) of embryos originating from sperm with centrosomal abnormalities revealed higher rates of aneuploidy, supporting a potential role of the paternal centrosome in ensuring accurate mitosis and proper early embryonic development [62]. Collectively, these studies indicate that centrosomal dysfunction can lead to irregular cleavage patterns, delayed cell divisions, increased fragmentation, and embryonic arrest, ultimately compromising blastocyst viability and implantation potential.

### 3.9. The Role of Centrosomes in Embryo Aneuploidy

During cell division, meiotic or mitotic errors may alter the number of chromosomes, resulting in aneuploid embryos. Although numerous studies have demonstrated the maternal origin of aneuploidy, knowledge of the paternal contribution remains limited. Given that the oocyte lacks centrioles, dysfunction in sperm centrioles can lead to improper spindle formation, cytokinesis failure, and defective chromosomal segregation, resulting in embryonic aneuploidy [63]. Recent evidence suggests that errors contributing to embryonic aneuploidy may arise as early as the zygote stage. Cavazza et al. showed that the unification of parental genomes is an error-prone process dependent on genome clustering at the pronuclear interface, which facilitates efficient chromosome capture and is associated with reduced segregation errors. Centrosomes, together with microtubules and dynein, contribute to this process, and their position may influence genome unification and chromosome segregation [51]. Kang and Rosenwaks emphasized the essential role of centrosomes in chromosomal segregation by comparing developmental outcomes in digynic and dispermic triploid embryos. In dispermic zygotes, fertilization by two sperm introduces two paternal centrosomes, leading to tripolar spindle formation, abnormal chromosomal segregation, and increased aneuploidy. In contrast, digynic zygotes, which retain an extra maternal chromosome set due to failure of second polar body extrusion, rely on a single sperm-derived centrosome to organize a bipolar spindle. This configuration is associated with more accurate chromosomal segregation and a lower incidence of mosaicism during early embryonic development [64]. Moomjy et al. investigated the impact of sperm integrity on chromosomal stability. Oocytes injected with dissected sperm components exhibited high rates of chromosomal mosaicism compared to those injected with intact spermatozoa. FISH analysis confirmed a high frequency of chromosomal abnormalities, with only a minority of embryos displaying a normal diploid complement. These findings support a link between impaired sperm centrosomal integrity and defective chromosome segregation during early embryogenesis [61]. Research has investigated the relationship between sperm quality and centrosome function, focusing on how defects in centrosome activity contribute to errors in mitotic spindle formation, leading to aneuploidy or mosaicism. In a case report, embryos derived from an OAT patient showed a high rate of chromosomal abnormalities, including haploidy, polyploidy, and chaotic mosaicism as revealed by FISH analysis. These alterations were likely related to centrosomal dysfunction affecting spindle formation and chromosome segregation. In contrast, embryos generated with donor sperm were chromosomally normal, supporting a role for sperm centrosomal integrity in chromosomal organization [65]. A similar study by Chatzimeletiou et al. investigated aneuploid embryo formation in a patient with OAT. All analyzed embryos exhibited chromosomal abnormalities, including polyploidy and post-zygotic chromosomal missegregation. These abnormalities were associated with defective spindle organization and uneven chromosome segregation, likely resulting from cytokinesis failure and aberrant centrosomal distribution [66]. Consistent with this, two other studies have highlighted a potential connection between poor sperm quality and embryo mosaicism. In one study, 340 cycles of PGT-A were examined, showing that reduced sperm quality was associated with a higher incidence of mosaic embryos, although no significant differences were found in the rates of euploid or aneuploid blastocysts [67]. Similar findings were reported by Kahraman et al., who analyzed 326 PGT-A cycles in patients with altered sperm parameters. They observed a high incidence of mosaicism as well as abnormal morphokinetic development, particularly in cases where testicular spermatozoa were used [68]. In a study, researchers investigated a case of recurrent embryo aneuploidy occurring in both natural and assisted reproduction, focusing on the male factor contribution. Although standard semen analysis revealed normal volume and pH, it also showed reduced progressive motility (18–25%) and poor morphology (3–4% normal forms). Immunofluorescence for tubulin revealed that 83% of the patient’s sperm exhibited two centriolar spots, indicating the persistence of both proximal and distal centrioles. However, centrin 1, a crucial protein for centrosomal function, was either mislocalized or absent in the majority of sperm, suggesting potential centrosomal dysfunction. TEM confirmed these structural abnormalities, revealing elongated centriolar adjuncts, indicative of incomplete sperm maturation. Additionally, FISH analysis showed increased frequencies of chromosomal anomalies, including disomy for chromosomes 9 and 18, as well as diploidy [63]. Cheung et al. investigated centrosomal defects in five couples who underwent multiple ICSI cycles but failed to obtain euploid embryos. Although immunofluorescence analysis showed the presence of the centrosome in 45.9% of spermatozoa, genetic testing identified mutations in *HAUS1*, essential for centrosome integrity, and in *KIF4A* and *XRN1*, which are involved in microtubule stabilization. Notably, one couple who underwent a subsequent ICSI cycle using microfluidic sperm selection achieved a clinical pregnancy with euploid embryos, suggesting that selecting sperm with improved genomic and centrosomal integrity may help overcome developmental barriers in selected cases [20].

### 3.10. Gene Expression Profiles Associated with the Centrosome

Proper expression of centrosome-related genes is critical for sperm development, supporting processes such as centriole assembly, flagellar formation, and the maintenance of structural integrity required for normal sperm function. Disruption of these processes has been strongly linked to male infertility [19,40,69,70]. Among these, *CEP135*, *CEP112*, *CEP128*, *CEP78*, *SPATC1L*, *CCDC146*, *AK7*, and *DZIP1* play key regulatory roles. Genetic mutations or altered expression of these genes are associated with disorders such as multiple morphological abnormalities of the sperm flagella (MMAF) and acephalic spermatozoa syndrome. MMAF is a severe flagellar defect in which sperm tails may be coiled, shortened, absent, or structurally abnormal, resulting in markedly reduced motility and fertility [70]. In contrast, acephalic spermatozoa syndrome, a rare and severe form of teratozoospermia, involves the formation of sperm cells lacking heads. Whole-exome sequencing (WES) performed on 22 infertile men affected by this condition led to the identification of compound heterozygous mutations (c.910C>T:p.Arg304Cys and c.994G>T:p.Glu332X) in the *SPATC1L* gene in one patient. This gene was found to be specifically expressed at the head–tail junction of elongating spermatids, a region essential for maintaining sperm structural integrity. Its deficiency disrupts sperm morphology and negatively impacts early embryonic development. In this case, although fertilization was achieved in all four ICSI cycles, none of the resulting embryos progressed to the blastocyst stage or were suitable for transfer [69]. The loss of function variants in *CEP112* have been implicated in acephalic spermatozoa. In a study involving two infertile patients, mutations in *CEP112* were identified. One patient presented with a homozygous variant in exon 5, c.496C>T:p.(Arg166X) while the other carried two mutations in exon 20, c.2074C>T:p.(Arg692Trp) and c.2104C>T:p.(Arg702Cys). Western blot analysis demonstrated robust CEP112 expression in control samples, while no detectable protein was observed in the first patient, and a markedly lower level was seen in the second. In addition, immunofluorescence analysis revealed that *CEP112* is typically localized in the centrioles of normal sperm. In contrast, it was scarcely detectable in the sperm head and flagella of the first patient and only faintly present in the second [71]. More recently, *CEP112* variants have been associated with oligoasthenoteratozoospermia. Bi-allelic variants in *CEP112* (NM_001199165.4, NP_001186094.1), identified in two unrelated individuals, were consistent with autosomal recessive inheritance and were associated with severely reduced sperm concentration, complete asthenozoospermia, and normal DNA integrity. Morphological and ultrastructural analyses revealed flagellar abnormalities and disruption of the canonical “9 + 2” microtubule arrangement, loss or misalignment of outer dense fibers, and defects in mitochondrial sheath. Sperm neck abnormalities, including incomplete or absent centrioles, further highlight the role of *CEP112* in centrosome integrity. These alterations were associated with poor ICSI outcomes, resulting in impaired embryonic development and miscarriage [72]. Another study investigated the genetic causes of severe asthenoteratospermia with MMAF in two patients from consanguineous families. Both exhibited severe flagellar defects, with absent flagella in most spermatozoa and markedly reduced motility and concentration. Between the two patients, one carried a *DZIP1* missense mutation (c.188G>A, p.Arg63Gln) and the other a stop-gain mutation (c.690T>G, p.Tyr230*). In addition, immunofluorescence staining for the centriolar protein Centrin 1 revealed that the proband’s spermatozoa exhibited abnormal centrosomes, characterized by the absence of a distinct centriolar signal or the presence of more than two centriolar dots. These mutations resulted in DZIP1-impaired centrosomal function and flagellar biogenesis, evidenced by abnormal centriole organization and absent flagella [73]. The gene *CEP135* is essential for the proper formation of centrioles, in particular, central pair assembly [70]. Altered expression has been linked to microtubule disorganization, which can contribute to the development of the MMAF phenotype. Sha et al. reported a homozygous mutation in *CEP135* (c.A1364T:p.D455V) in a patient exhibiting the MMAF phenotype. This mutation was associated with abnormal aggregation in both centrosomal and flagellar structures, indicating a potential disruption of centriole assembly. Despite ICSI, no pregnancy was achieved following embryo transfer [74]. WES performed in a cohort of 167 men with MMAF identified bi-allelic truncating variants in the *CCDC146* gene in two unrelated patients, including a nonsense mutation (c.1084C>T) and a frameshift deletion (c.2112del), both predicted to result in the absence or production of non-functional proteins. Functional analyses showed that, in spermatozoa, *CCDC146* is not localized to centrioles, unlike in somatic cells, but is instead present along the axoneme at the level of microtubule doublets. Its absence disrupted multiple microtubule-based structures, including the manchette, head–tail coupling apparatus, and axoneme [75]. Similarly, whole-exome sequencing identified a novel homozygous nonsense variant in the *AK7* gene (c.1153A>T; p.Lys385*) in two infertile siblings presenting with asthenoteratozoospermia and MMAF. Immunoblotting and immunofluorescence analyses demonstrated a nearly complete absence of *AK7* protein in patient spermatozoa, which also exhibited mitochondrial dysfunction and severe flagellar abnormalities, including axonemal defects and mitochondrial alterations [76]. Recent studies have emphasized the importance of *POC1A* and *POC1B* in maintaining centrosome function during human spermatogenesis. Mutations in *POC1A* have been associated with defective spindle formation, impaired meiosis, and aneuploidy, ultimately leading to compromised sperm production. Loss of *POC1A* function may lead to supernumerary centrioles and impaired Golgi stack organization, thereby disrupting intracellular trafficking. Similarly, mutations in *POC1B*, typically deletions or missense variants affecting exons 6 or 7, result in the loss of association between the basal body and the primary cilium. These alterations produce immotile sperm with disrupted axoneme architecture, further contributing to male infertility [19]. A homozygous missense variant in *CEP128* (c.665G>A [p.R222Q]) was found in two infertile siblings, associated with severely reduced sperm concentration, motility, and abnormal morphology, particularly affecting the head–tail junction. TEM showed marked ultrastructural defects, with partial formation of the proximal and distal centrioles at the connecting piece. Compared to control sperm with an intact “9 + 2” axonemal structure, patient sperm exhibited disorganized or absent central-pair microtubules, malformed outer dense fibers, and disrupted microtubule doublets across the flagellum. These structural abnormalities are consistent with severe centrosomal dysfunction. Despite several ICSI attempts, both fertilization and embryo development failed, likely due to defects in centrosome-dependent mechanisms required during the earliest stages of embryogenesis [77]. Another study of a consanguineous family revealed a novel homozygous splicing mutation (c.1069 + 1G>A) in the *CEP78* gene as the likely cause of OAT and associated hearing and vision impairments. The mutation was found in a 28-year-old male with seven years of infertility. His brother, who carried the same homozygous mutation, was also infertile, whereas unaffected relatives were heterozygous carriers. Affected individuals showed severely reduced sperm count, motility, and abnormal morphology with flagellar defects. TEM revealed structural abnormalities of the axoneme and centrioles, including disruption of the “9 + 2” architecture and defective proximal and distal centrioles [78]. Yu et al. identified *HYDIN* mutations in two patients with asthenoteratozoospermia, a gene associated with Primary Ciliary Dyskinesia. One patient had two heterozygous *HYDIN* missense variants (c.9507C>G [p.N3169K] and c.14081G>A [p.R4694H]), while the other had two heterozygous splicing variants (c.5969-2A>G and c.6316+1G>A). In the latter patient, TEM revealed that most sperm lacked the central pair of microtubules, showing “9 + 0” or “9 + 1” axonemes instead of the normal “9 + 2” microtubule structure. Immunofluorescence and Western blotting revealed centrosomal abnormalities, with altered localization and reduced expression of centrin 1. Interestingly, the use of ICSI in this patient successfully overcame the infertility phenotype, resulting in a pregnancy [79]. In another study, two unrelated patients with oligoasthenoteratozoospermia were found to have two bi-allelic mutations in the *CEP70* gene. One patient harbored compound heterozygous variants, c.1058C>G (p.Gly353Ala) and c.1059_1063del (p.Trp354Thrfs*14), whereas the second patient carried a homozygous frameshift variant: c.1842dupT (p.Pro615Thrfs*14). Additional analyses using immunofluorescence and Western blotting showed that *CEP70,* normally localized to the sperm neck and acrosome, was almost absent in the patient’s spermatozoa, with Western blot results also suggesting protein degradation. Morphological evaluation and TEM demonstrated severe abnormalities, including round or amorphous sperm heads, irregular tail structures and incomplete or disorganized “9 + 2” axonemal microtubule structures [80]. A detailed investigation of seven couples presenting with recurrent implantation failure, despite completing eight ICSI cycles and multiple embryo transfers, revealed that none initially achieved a clinical pregnancy. Although centrosome evaluation showed structurally normal centrioles in 53.1 ± 13% of spermatozoa, genetic analysis identified an average of 44 ± 13 mutations per patient in genes essential for centrosome and microtubule organization, including *MAP1S*, *PLK4,* and *SUPT5H* [20].

### 3.11. Centrosomal Alterations in Patients with Globozoospermia, Kartagener’s Syndrome, and Dysplasia of Fibrous Sheath

Globozoospermia, Kartagener’s syndrome, and dysplasia of fibrous sheath (DFS) are rare forms of male infertility, each associated with distinct structural and functional abnormalities in sperm. Globozoospermia is a rare form of teratozoospermia in which sperm exhibit a rounded head morphology and fail to develop acrosomes, thereby lacking the enzymes required for zona pellucida penetration and successful fertilization. This condition is also associated with abnormalities in chromatin condensation and impaired oocyte activation [17,81,82]. Nakamura et al. analyzed sperm aster formation and male pronucleus formation in a patient with globozoospermia compared to two fertile controls. The study used ICSI on bovine oocytes followed by immunofluorescence analysis. Results revealed a significantly lower rate of sperm aster formation (15.8%) and male pronucleus formation (31.0%) in the patient compared to the controls (57.9% and 92.5%, respectively). Although AOA with ethanol improved the rate of male pronucleus formation to 84.9%, the sperm aster formation rate remained low at 32.3% [81]. Additionally, Moretti et al. analyzed centrin 1 in sperm from one globozoospermic patient and three fertile men using immunofluorescence. In globozoospermic sperm, centrin 1 displayed abnormal localization around the head, while in fertile sperm it appeared as two distinct foci at the nuclear base, representing the proximal and distal centrioles [82]. Palermo et al. evaluated a single patient diagnosed with globozoospermia. The patient had undergone two previous ICSI cycles, both of which resulted in fertilization failure, despite the application of AOA. Centrosomal integrity was examined using immunofluorescence staining for centrin 1. In fertile sperm samples, centrosomes are typically observed in 60–80% of spermatozoa. In this case, centrosomal presence was borderline normal at 57%, suggesting a potential compromise in centrosomal functionality. However, when a proprietary assisted gamete activation protocol, targeting both the male gamete and the oocyte, was applied in a subsequent ICSI cycle, calcium ionophore activation led to the development of two viable embryos, both of which resulted in healthy births [83]. DFS is a rare form of teratozoospermia characterized by structural abnormalities extending from the midpiece to the tail, often resulting in immotile spermatozoa. These deformities severely impair sperm motility and frequently lead to fertilization failure [17]. In a heterologous ICSI study using bovine oocytes, sperm from a fertile donor and a DFS patient were analyzed. Aster formation was frequent in motile sperm from fertile donors (82.6%, 38/46), reflecting intact centrosomal function. However, oocytes injected with DFS sperm rarely exhibited aster formation (9.5%, 4/42). In comparison, dead sperm from the same donors failed to form asters and did not induce oocyte activation (0/37). When these sperm were treated with dithiothreitol (DTT) before ICSI and followed by paclitaxel (Taxol™) treatment post-ICSI, aster formation significantly improved to 70.1% (40/57). Conversely, sperm from a patient with DFS, characterized by profound centrosomal dysfunction and severely reduced centrin expression (<2% of normal), showed only minimal improvement, with aster formation observed in just 7.7% (3/39) of oocytes. While DTT and Taxol can effectively restore centrosomal function in compromised but structurally intact sperm, they are insufficient to rescue the severe centrosomal defects associated with DFS [84]. Moretti et al. investigated a patient with DFS using immunocytochemical staining for centrin 1, which was present in 67% of spermatozoa, but only 13% exhibited normal localization. In most cases, its positioning was altered or misaligned, sometimes appearing bent. In some sperm, four distinct centrin 1 signals were observed, likely indicating centriole duplication. Additionally, fragmented centrin 1 signals were detected at the centriolar region, frequently extending along the short and thick tail [85]. Kartagener’s syndrome, a genetic disorder under primary ciliary dyskinesia, causes immotile sperm due to axonemal defects. Von Zumbusch et al. evaluated whether sperm centrosomal function remains intact in two affected individuals and whether ICSI can bypass motility defects to achieve fertilization and embryo development. Despite severe ultrastructural abnormalities, including the absence of dynein arms and central fibrils, successful fertilization and embryo development were achieved in both patients, resulting in healthy births. These findings indicate that, although motility is impaired, centrosomal function may remain intact, supporting the use of ICSI in these patients [18].

### 3.12. Current and Emerging Treatments for Sperm Centrosomal Defects

Recent advancements in reproductive medicine have underscored the pivotal role of the sperm centrosome in embryonic development. Addressing centrosomal dysfunction presents a valuable opportunity to improve clinical outcomes, particularly for patients facing recurrent fertilization failure, embryo arrest, and increased aneuploidy rates following ICSI. One promising approach to overcoming defective sperm centrosomal function in humans involves the transplantation of a functional sperm centrosome. Studies suggest that microinjecting an isolated and normal centrosome into the oocyte at the time of ICSI may compensate for defective sperm centrosomal function [30]. Van Blerkom and Davis demonstrated that centrosomes can be successfully isolated and reintroduced into human oocytes, where they support microtubule organization and mitotic spindle formation. The researchers isolated sperm centrosomes from fertile donors, separated them from the sperm heads, and microinjected them into a total of 24 MII oocytes collected from seven patients. The oocytes were then cultured for up to 18 h, and microtubule organization was analyzed using immunofluorescence and confocal microscopy. Results showed that the injected centrosomes successfully nucleated maternal tubulin to form well-developed microtubule asters in the oocytes, confirming that sperm centrosomes are functionally active post-transfer. This was further validated by the absence of microtubule asters in control oocytes that did not receive a centrosome injection [41]. Further indirect support for the feasibility of centrosome-based interventions comes from the previously mentioned case by Emery et al., in which successful fertilization and embryo development were achieved despite the use of mechanically separated sperm components. Although this did not constitute a direct centrosome transfer, the findings suggest that maintaining the integrity and correct positioning of the sperm midpiece may preserve essential centrosomal activity, thereby sustaining normal developmental potential. This observation highlights the clinical promise of approaches aimed at restoring centrosomal function and suggests that, with further technical refinement, centrosome donation could evolve into a viable therapeutic option for patients with severe centrosomal dysfunction [59]. However, before clinical implementation, additional research is needed to ensure the safety and efficacy of centrosome transfer, particularly regarding potential risks of multipolar spindle formation and chromosomal instability. Another avenue under investigation is the chemical restoration of sperm centrosomal function. Experimental treatments have explored the combined use of DTT, which breaks disulfide bonds and helps unravel the sperm centrosome, and Taxol, a microtubule-stabilizing agent that promotes polymerization. Nevertheless, despite showing some promise, these interventions have not demonstrated consistent efficacy, especially in cases of severe centrosomal dysfunction such as DFS [84].

## 4. Discussion

This systematic review highlights the pivotal role of the human sperm centrosome across multiple stages of reproduction, from fertilization and early embryonic development to its potential contribution to embryonic aneuploidy, as well as its involvement in some forms of male infertility [31]. Disruptions in centrosomal function, including defective microtubule organization and impaired aster formation, can compromise fertilization and early embryonic development [13,14,30,52,53]. Simerly et al. provided early evidence highlighting the essential role of centrosomal integrity in fertilization, showing that oocytes exposed to nocodazole, a compound that disrupts microtubule formation, were unable to generate sperm aster microtubules and failed to fertilize. However, fertilization was successfully achieved when donor sperm was used with oocytes from the same cohort [2]. In line with these findings, Van Blerkom and Navara et al. reported that abnormalities in the sperm centrosome may impede pronuclear migration and compromise accurate spindle assembly [29,52,56,57,59]. Studies in human embryos have shown that normal centrosome duplication and spindle organization are associated with regular cleavage patterns, whereas centrosomal defects have been linked to abnormal or delayed divisions [15,60]. Supporting this observation, micromanipulation studies demonstrated that although oocytes injected with dissected spermatozoa can undergo fertilization and initiate cleavage, they frequently exhibit abnormal mitotic divisions and increased chromosomal mosaicism, likely due to defective spindle assembly [61]. Similarly, evidence from heterologous ICSI models by Terada et al. and Yoshimoto-Kakoi et al. showed that reduced sperm aster formation is associated with lower cleavage rates, indicating that centrosomal activity directly influences early embryonic division dynamics [46,47,61]. Moreover, Chatzimeletiou et al. showed that abnormal centrosomal distribution in cleavage-stage embryos can result in defective spindle formation, asymmetric centrosome allocation, and cytokinesis failure, leading to polyploidy and post-zygotic chromosome missegregation. These defects were associated with delayed or arrested embryonic development [7,45,46]. In line with this, Tarozzi et al. reported that centrosomal dysfunction correlates with irregular cleavage patterns, impaired division kinetics, increased fragmentation, and elevated rates of aneuploidy [62]. In many cases, these abnormalities are accompanied by chromosomal instability, including aneuploidy and mosaicism, driven by impaired centrosomal function and defective spindle assembly. This is exemplified by a case of recurrent embryo aneuploidy, in which altered localization of centrosomal proteins and structural abnormalities of the centrioles were associated with increased rates of chromosomal anomalies such as disomy and diploidy, suggesting impaired chromosome segregation during early embryogenesis [63]. Consistent with these findings, Cheung et al. reported centrosomal defects and mutations in centrosome-related genes in couples with repeated ICSI failure and absence of euploid embryos. Notably, in one case, the selection of spermatozoa with improved structural integrity using microfluidic techniques was associated with successful pregnancy and the development of euploid embryos, further supporting a functional link between centrosomal integrity and clinical reproductive outcomes [20]. Many centrosomal proteins play critical roles in supporting microtubule organization. Centrin, which is abundantly expressed in mature centrioles, has been found at significantly lower levels in sperm from individuals with oligoasthenozoospermia [10]. Similarly, γ-tubulin, a core component involved in microtubule nucleation, is also diminished in sperm from infertile men [7,11,20,63,67,68]. Genetic alterations affecting centrosome-associated proteins, including *CEP135* and *CEP112*, have been linked to pronounced abnormalities in sperm structure. Specifically, alterations in *CEP135* have been linked to MMAF, while defects in *CEP112* are implicated in the acephalic spermatozoa phenotype [71,74]. Likewise, pathogenic variants in *CEP128* have been connected to fertilization failure and early developmental arrest of embryos [77]. In addition, some forms of male infertility caused by mutations in *DZIP1*, *HYDIN*, and *CEP78* disrupt centriole biogenesis, further emphasizing the critical role of centrosomal gene defects in reproductive failure [73,78,79]. Therapeutic strategies under investigation for sperm centrosomal abnormalities encompass both centrosome transfer and chemical-based treatments. Experimental microinjection of isolated centrosomes into oocytes has demonstrated the capacity to reestablish microtubule aster formation and promote fertilization [30,41]. Similarly, chemical agents such as DTT and Taxol have also been investigated to enhance sperm aster formation; however, their efficacy appears limited in severe abnormalities like DFS defects [84]. While these methods show potential, additional research is required before they can be reliably implemented in clinical settings. Although centrosomal integrity is essential for reproductive success, it is rarely assessed in standard clinical practice. Therefore, integrating centrosome-focused diagnostics and therapeutic strategies into ART may improve outcomes in patients with centrosome-related infertility and support more individualized approaches to fertility care. Future research should focus on refining techniques such as centrosome transplantation, enhancing the efficacy of chemical interventions, and developing standardized diagnostic tools that can guide tailored fertility treatments.

## 5. Limitations

This review focuses on human sperm centrosome biology; therefore, some relevant research from non-human contexts may have been excluded, potentially limiting the inclusion of certain functional insights. However, selected experimental data from animal models were included when part of studies that also reported human data and provided essential support or validation. Such approaches were considered when they offered functional insights that cannot be directly obtained in humans due to ethical and technical limitations. For example, heterologous ICSI assays using bovine or rabbit oocytes and ex vivo systems such as Xenopus laevis egg extracts have been widely used to evaluate sperm centrosomal function, including aster formation and spindle assembly. Nevertheless, due to biological differences between species and experimental systems, data from animal or ex vivo models should be interpreted with caution when applied to human reproductive biology. A formal risk-of-bias assessment was not performed due to the heterogeneity of the included study designs, and this should be considered when interpreting the findings of this review. Furthermore, many of the included studies are observational or based on limited sample sizes and therefore do not allow the establishment of causal relationships between centrosomal alterations and reproductive outcomes.

## Data Availability

No new data were created or analyzed in this study.

## Supplementary Materials

The following supporting information can be downloaded at: https://www.mdpi.com/article/10.3390/life16060921/s1; Table S1: Characteristics, study design, and main findings of included studies.

## Abbreviations

The following abbreviations are used in this manuscript:

AB	Abnormal
Anti-MSP	Anti-mitotic spindle protein
AOA	Artificial oocyte activation
ART	Assisted Reproductive Technology
BP	Bipolar
CA	Centriolar adjunct
CETN	Centrin
DC	Distal centriole
DFS	Dysplasia of the fibrous sheath
DTT	Dithiothreitol
FISH	Fluorescence in situ hybridization
FRAC	Fluorescence-Based Ratiometric Analysis of Sperm Centrioles
G1-PCC	G1-phase premature chromosome condensation
HP1γ	Heterochromatin Protein 1γ
ICSI	Intracytoplasmic sperm injection
IMS	Idiopathic male sterility
MII	Metaphase II
MMAF	Multiple morphological abnormalities of the sperm flagella
MPF	Mitosis-promoting factor
MTOC	Microtubule-organizing center
NS	No structure
OA	Oligoasthenozoospermia
OAT	Oligoasthenoteratozoospermia
PCM	Pericentriolar material
PC	Proximal centriole
PGT-A	Preimplantation genetic testing for aneuploidy
TEM	Transmission electron microscopy
WES	Whole-exome sequencing
WOS	Web of Science
γ-TuRC	γ-tubulin ring complex
